# Glutathione Transferase as a Potential First-Tier Biomarker of Environmental Pollution in Workers and Residents of Umbria Region, Italy

**DOI:** 10.3390/antiox15060741

**Published:** 2026-06-11

**Authors:** Sara Notari, Fiorella Faienza, Giovanna Tranfo, Anna Maria Caccuri, Giorgio Ricci, Giorgia Gambardella

**Affiliations:** 1 Department of Chemical Sciences and Technologies, University of Rome ‘Tor Vergata’, Via della Ricerca Scientifica 1, 00133 Rome, Italy; sara.notari@uniroma2.it (S.N.); fiorella.faienza@uniroma2.it (F.F.); caccuri@uniroma2.it (A.M.C.); 2 Department of Occupational Medicine, Epidemiology, Occupational and Environmental Hygiene, INAIL Research, Via Fontana Candida 1, Monte Porzio Catone, 00078 Rome, Italy; giovanna.tranfo@gmail.com

**Keywords:** environmental pollution, erythrocyte glutathione transferase, oxidative stress, occupational exposure monitoring, human biomonitoring

## Abstract

Erythrocyte glutathione transferase (e-GST) and oxidized human serum albumin (HSAox) were used as biomarkers to assess the environmental health of a foundry and a ceramics factory located in the Umbria region (Italy). While HSAox level indicates that no relevant oxidative stress is present in workers of either company, a distinct overexpression of e-GST, also present in non-workers residents, suggests exposure to broad environmental stressors that may be attributed to common sources. A more careful analysis of different job sectors and the effect of filtering facepieces indicates that the workers exposed to solvents show the highest e-GST expression, while different pollutants (possibly dust and/or metal fumes) seem to be present in other areas of the foundry as well as in external areas. In the ceramic company, the high e-GST activity reveals the presence of other contaminants which can be efficiently blocked by filtering facepieces and gloves together. Intriguingly, a cohort of monks (*n* = 14) residing in a monastery adjacent to the foundry exhibited e-GST activities significantly lower than those observed in non-worker residents, suggesting that specific lifestyle or localized environmental factors may influence exposure levels, a finding that warrants further targeted research.

## 1. Introduction

In spite of growing concerns and strict legislation, the impact of toxic substances that many workers may come into contact with on human health remains a serious and urgent workplace problem. A few of these substances, along with their health effects, are well known. However, many others remain unknown because hundreds of new organic compounds enter every year into various activities, and only some of them have an established medium- and long-term impact on human health. Consequently, the development of first-tier biomarkers capable of detecting the early presence of toxic substances in both occupational settings and residential areas is of paramount importance.

Erythrocyte glutathione transferase (e-GST) and oxidized human serum albumin (HSAox) are good candidates for this role. Glutathione transferase (GST) is a multifunctional superfamily of detoxifying enzymes capable of protecting all living organisms from exposure to dangerous contaminants [1]. They catalyze the conjugation of glutathione (GSH) to many toxic compounds with an electrophilic center, making them less reactive, more soluble, and more easily eliminated from cells. Other detoxifying functions are performed by GST acting as ligandins, binding a wide range of toxins and promoting their excretion from the cell [2]. Moreover, some GSTs also possess a distinct non-catalytic function, serving as negative regulators of key cellular signaling pathways. This non-enzymatic protection is exemplified by GSTP1-1, which inhibits the mitogen-activated protein kinase (MAPK)/c-Jun N.terminal kinase (JNK) signaling cascade through the sequestration of JNK and TNF receptor-associated factor 2 (TRAF2), thereby decreasing oxidative stress-induced apoptosis [3,4,5,6]. In mammals, GST isoenzymes are present in almost all tissues subdivided into seven gene independent classes [7,8]. The erythrocytes contain almost exclusively a single isoenzyme, i.e., GSTP1-1 (e-GST), also identified as GSTPi. It has been shown that this specific enzyme is overproduced when humans or animals are exposed to toxic substances, making it a potential biomarker for environmental pollution in both human populations and livestock [9]. Relevant overexpression has also been observed in subjects with kidney diseases, clearly due to an increase in circulating endogenous toxins [10]. A second useful candidate biomarker, i.e., HSAox, can be used to assess possible exposure to compounds which trigger oxidative stress [11]. However, this oxidized protein represents a short-term indicator of oxidative insult while the activity of e-GST may be related to exposure of contaminants for over two to three months (corresponding to the half-life span of the erythrocytes) [12,13].

In the present study, we present a few data concerning e-GST activities in two different working environments: a steel industry and a ceramics factory, both located in the Umbria region (Italy) within a few kilometers of each other. A third monitored environment was a Catholic convent located near the foundry. The e-GST activities were compared with those of non-working individuals residing in the same area. Our findings provide a preliminary indication of elevated chemical stress consistent with widespread environmental factors in both regions. No evident pollution triggering oxidative stress was found in any tested subjects, at least when using oxidized albumin as a biosensor. A surprising finding is that the monks exhibit e-GST activity approaching that of an uncontaminated population. A detailed study was also conducted to determine the correct methods for storing blood samples for accurate e-GST determination.

## 2. Materials and Methods

### 2.1. Chemical and Reagents

Glutathione (GSH), 1-chloro-2,4-dinitrobenzene (CDNB), ethylenediaminetetraacetic acid (EDTA), cystamine, and 5,5′-dithiobis (2-nitrobenzoic acid) (DTNB) were purchased from Sigma-Aldrich (St. Louis, MO, USA).

### 2.2. Study Design

The study was divided into two distinct phases: a methodological stability assessment and an occupational exposure monitoring. Initially, an e-GST stability study was performed on blood samples from healthy subjects (*n* = 10) at the Transfusion Medicine Section of the Policlinico Tor Vergata (PTV), under the approval of the PTV Ethical Committee. Building on these foundational data, a second study focused on contaminant exposure, by monitoring e-GST and human HSAox, was conducted in the Umbria region, involving different groups of volunteers (*n* = 124) comprising residents (*n* = 28), monks (*n* = 14), foundry workers (*n* = 68), and ceramists (*n* = 19) (Table 1). The residents group (*n* = 28) consisted of individuals living in the immediate vicinity (within a 2 km radius) of the industrial sites (foundry and ceramics factory). These subjects were not occupationally exposed to the industries but shared the same environmental air and water sources as the workers. Inclusion criteria for residents were residency in the area for at least 5 years, non-smoking status, and no history of work in the metallurgical or ceramic sectors. All the enrolled workers had no significant pathologies and were non-smokers. For both studies, blood samples were collected via venipuncture in K3-EDTA tubes (Vacutainer, BD, Plymouth, UK) and immediately processed: one aliquot was centrifuged (3700 g for 3 min) to obtain plasma and stored at −20 °C for HSAox preservation [14], while the whole blood was stored at +4 °C for e-GST analysis. All the samples were analyzed within 24 h of collection. Ethical oversight for the second phase was provided by the Ethical Committee “CET Lazio Area 2” (Protocol: “BRIC ID 52”, ID:113.24 CET2 ptv). All procedures were conducted in strict accordance with the Declaration of Helsinki and the International Code of Ethics for Occupational Health Professionals (ICOH), ensuring data was processed in aggregate form to maintain participant anonymity. The control group used in this study was determined in our previous study of Fabrini and coworkers [13]. This group consisted of 400 healthy volunteers recruited from the Transfusion Medicine Section of the Policlinico Tor Vergata. The participants of the control group followed the criteria: being non-smokers, having no history of occupational exposure to industrial pollutants, and residing in urban or rural areas with no known significant environmental contamination. These subjects were used to establish the baseline e-GST activity (5.3 ± 0.5 U/g Hb for males and 5.9 ± 0.4 U/g Hb for females), against which all other Umbrian cohorts were compared.

### 2.3. e-GST Activity Assay

e-GST activity was measured using a spectrophotometric assay at 340 nm (37 °C) by an Uvikon 941 Plus spectrophotometer (Kontron Instruments, Watford, UK). First, 40 μL of whole blood was diluted into 1 mL of Milli-Q water; this first dilution provokes erythrocytes hemolysis. For the second step, 100 μL of hemolyzed blood was diluted to a final volume of 1 mL of 0.1 M potassium phosphate buffer, pH 6.5, containing 1 mM GSH and 1 mM CDNB, according to Habig and coworkers [15]. Final data were reported as enzyme units (U) per gram of hemoglobin (Hb) (U/g Hb) [16]. One unit is the amount of enzyme that catalyzes the conjugation of one micromole of GSH to CDNB in 1 min at 37 °C.

### 2.4. e-GST Stability in Whole and in Hemolyzed Blood

Human blood samples (*n* = 10) with EDTA were aliquoted and stored at different temperatures: +25 °C, +4 °C, −20 °C, and −80 °C. In parallel, 40 μL aliquots of human blood (*n* = 10) were hemolyzed with 1000 µL of Milli-Q water and stored at +4 °C, −20 °C, and −80 °C. The storage temperature of +25 °C was not adopted for hemolyzed samples due to rapid protein deterioration. The e-GST activity in all blood samples stored at different temperatures was evaluated starting from the day of collection, and then at regular intervals from day 1 up to a maximum of 30 days, depending on the physical state and stability of the samples.

### 2.5. e-GST Activity in Worker, Monks and Residents in Umbria Country

Venous blood samplings were stored at +4 °C and analyzed within 24 h. The study population was stratified by sex, as this biomarker presents different basal values in males and females [17]. Moreover, results are shown according to the different tasks and workplace areas. Foundry and ceramic workers have at their disposal personal protective equipment (PPE) of the upper airways, namely class 2 or 3 filtering facepieces (FFP2 and FFP3), as well as gloves in the case of ceramists. Some workers wear both PPE, while others wear FFPs only or gloves only. In some cases, foundry employees, even if they are not identified as occupationally exposed, wear filtering facepieces when their workplaces are close to the production area of the foundry. As for the residents not working in the two workplaces considered, they do not wear any PPE.

### 2.6. HSAox in Worker, Monks and Residents in Umbria Country

The serum was obtained as previously described from the samples collected for e-GST analysis. The percent of oxidized HSAox was determined by subtracting the value of reduced HSA (HSAred) from the total HSA (g/dL) determined by routine protocol according to the standard procedure on an Architect instrument (Abbott Diagnostics Division, Abbott Park, IL, USA). The reduced HSAred cannot be easily evaluated using a direct spectrophotometric procedure; the single cysteine (Cys34) not involved in a disulfide bridge reacts very slowly with 5,5′-dithiobis (2-nitrobenzoic acid) (DTNB), a well-known thiol reagent [18]. A modified procedure based on the fast reaction of cystamine with Cys34 was adopted [14,19]. The released cysteamine, stoichiometric to the reduced Cys34, was determined with DTNB (Ɛ412 nm = 14.1 mM-1 cm-1of TNB) [18]. The assay was performed using a Kontron Uvikon 941 Plus spectrophotometer (Kontron Instruments, Watford, UK) at 412 nm (25 °C). Then, 50 μL of serum was diluted in 890 μL of potassium phosphate buffer 0.1 M pH 8.0, and then 50 μL of DTNB (50 μM final concentration) and 10 μL of cystamine (1 mM final concentration) were added to the solution. The final absorbance at 412 nm was recorded after 15 min of incubation. In this case, the study population was not stratified by sex because HSAox% does not depend on sex.

### 2.7. Statistical and Graphical Analysis

Data are reported as means ± standard deviation (SD) for the stability study, and as means ± standard error of mean (SEM) for the contaminant exposure study of Umbria region. One-way Anova was used to compare the e-GST activity variation relative to day 0 as well as to compare HSAox of each Umbria group to the control group. In both cases, Tukey’s post hoc test was applied for multiple comparisons. Regarding the e-GST activities of Umbria residents and workers, the resulting groups were smaller in size because the data were stratified by sex, job type, and PPE usage. For this reason, a Brown–Forsythe and Welch Anova test was performed. Moreover, to account for multiple comparisons, we applied Dunnett’s T3 multiple comparison test, a post hoc test recommended when the group sizes are small (*n* < 50) and the variances are not equal. In all analyses, a *p* ≤ 0.05 was considered statistically significant [20]. Graphics and result visualizations were obtained using GraphPad Prism software v9.5 (La Jolla, CA, USA).

## 3. Results

### 3.1. Stability of e-GST After Blood Withdrawal

**Figure 1:**
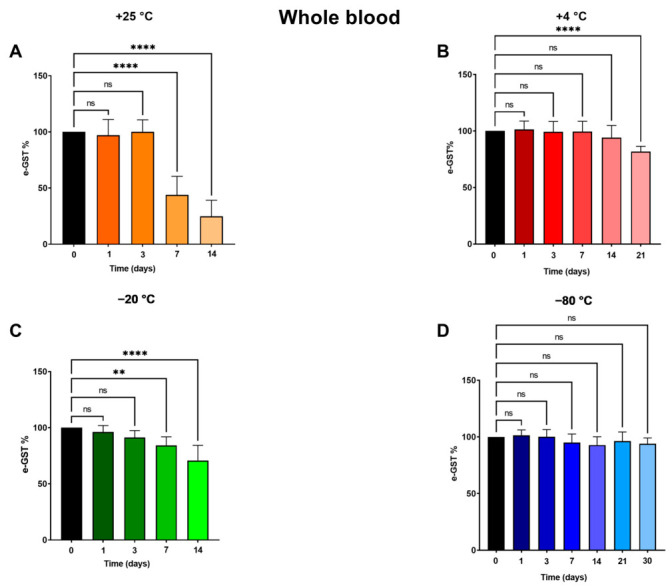
Stability of e-GST activity in human whole blood samples after storage at different temperatures. Whole blood stored at +25 °C (**A**), +4 °C (**B**), −20 °C (**C**), −80 °C (**D**). The e-GST activities (at days 1, 2, 3, 7, 14, 21, and 30) were normalized to the day 0 values (100%). The error bars represent the SD, ns (not significant), *p* ≤ 0.01 (**), and *p* ≤ 0.0001 (****).

**Figure 2:**
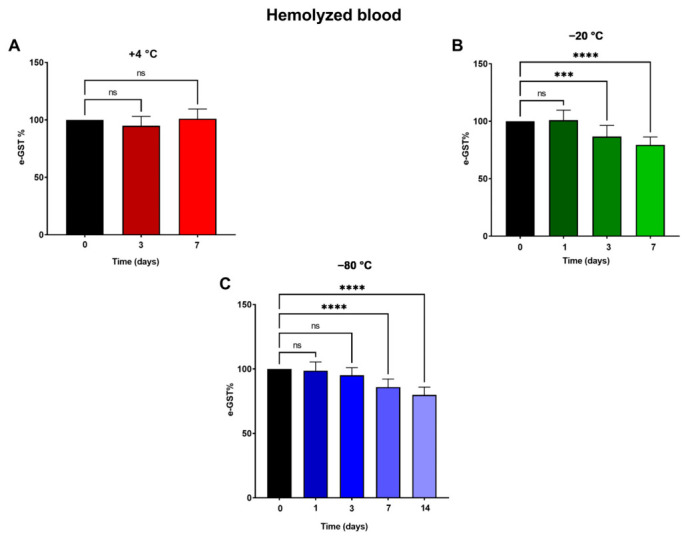
Stability of e-GST activity in human hemolyzed blood samples after storage at different temperatures. Hemolyzed blood stored at +4 °C (**A**), −20 °C (**B**), −80 °C (**C**). The e-GST activities (at days 1, 2, 3, 7, and 14) were normalized to the day 0 values (100%). The error bars represent the SD, ns (not significant), *p* ≤ 0.001 (***), and *p* ≤ 0.0001 (****).

As shown in Figure 1, a few different storage conditions of blood samples were adopted to assess the stability of the e-GST. Enzyme activity remained largely unchanged for up to 14 days when EDTA-treated blood samples were stored at 4 °C; however, at 25 °C, stability was maintained only for 3 days (Figure 1). After 7 days at 25 °C, more than 50% of the initial activity is lost. A surprising finding emerged during the storage of untreated blood at −20 °C because a progressive loss of activity occurs after a few days of storage. The remaining activity is reduced to 84% after 7 days, and to 71% after 14 days. Storage of blood at −80 °C allows the preservation of the original activity for at least 30 days. However, 20% of activity is lost after 7 days at −80 °C if the blood sample was hemolyzed before storage (Figure 2). A similar trend is observed for hemolyzed samples stored at −20 °C, while surprising stability was observed at 4 °C for up to 7 days (Figure 2A,B). Loss of activity was observed for whole blood at −20 °C, and the one observed at 25 °C after 14 days seems to be due to a conformational change in e-GST, with the G-site causing a loss of affinity for GSH. In fact, after these two different storage modalities, K_M_ for GSH appears largely increased (0.7 mM compared to 0.1 mM of the native enzyme). Meanwhile, K_M_ for the co-substrate CDNB is identical to that of the native GSTP1-1 (1 mM). This loss of affinity is likely dictated by the slow freezing rate of erythrocyte at −20 °C. It is well known that lower freezing rates result in larger ice crystals, interfering with the vitrification process. Consequently, this may irreversibly modify the conformation of the G-site, leading to the observed loss of affinity. These findings are in line with studies on vitrification and protein stability [21].

### 3.2. Oxidized Human Serum Albumin (HSAox) in All Individuals Enrolled in the Study

**Figure 3:**
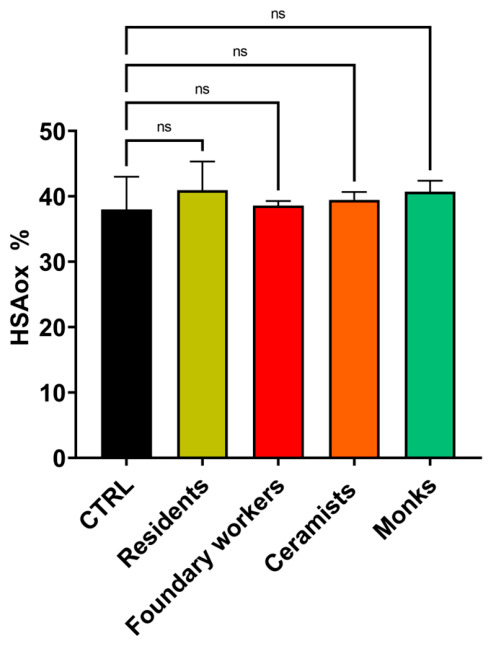
HSAox% levels in residents and workers. Level of HSAox% in the control group (black), residents (*n* = 28, yellow), foundry workers (*n* = 68 red), ceramists (*n* = 19, orange), and monks (*n* = 11, green). Error bars represent SEM, ns (not significant) compared to the control population.

A study conducted in the past by our group on 80 healthy individuals has allowed us to establish a reference interval for healthy subjects [22]. This corresponds to a percentage of HSAox equal to 38 ± 5%. The analyses conducted on the serum samples of all the subjects enrolled in this study (workers of foundry and ceramic compartments, residents and monks) show that the HSAox% values fall within the reference interval (Figure 3). An identical result was obtained, even after subdividing workers by tasks and exposure type, yielding identical results. Therefore, the analysis of HSAox levels in both the working and resident populations did not reveal significant oxidative modifications specifically to serum albumin.

### 3.3. e-GST Activity of Male Foundry Workers

**Figure 4:**
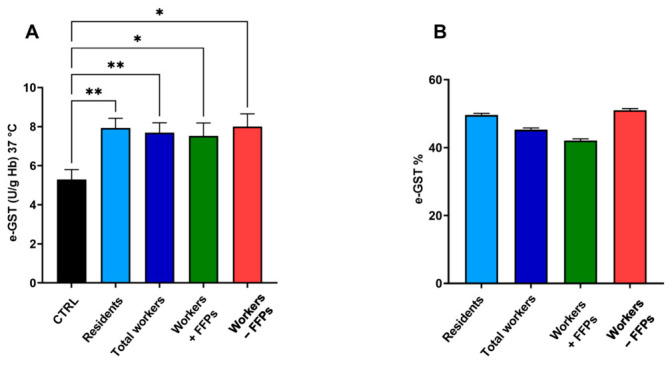
e-GST activity in male residents and foundry workers divided by FFPs usage. (**A**) e-GST enzyme activity in the male control group (black), residents (*n* = 13, light blue), total male workers (*n* = 34, blue), workers wearing FFPs (*n* = 22, green), and workers not wearing FFPs (*n* = 12). (**B**) Percentage increase of e-GST activity compared to the control (5.3 U/g Hb) in the group of residents (light blue), total male workers (blue), workers wearing FFPs (green), and workers not wearing FFPs (red). Error bars represent SEM, *p* ≤ 0.05 (*), *p* ≤ 0.01 (**) compared to the control population.

The e-GST activity of all male foundry workers was compared with that of the male residents (Figure 4). Surprisingly, both groups showed significantly higher e-GST activity (residents +50%, workers +45%) than that of the uncontaminated control group (5.6 U/g Hb) [17]. These data suggest some widespread environmental stressors present in the foundry as well as in the residential area. Interestingly, workers using FFPs only show a slightly lower expression of e-GST when compared to those not using this protection (Figure 4).

**Figure 5:**
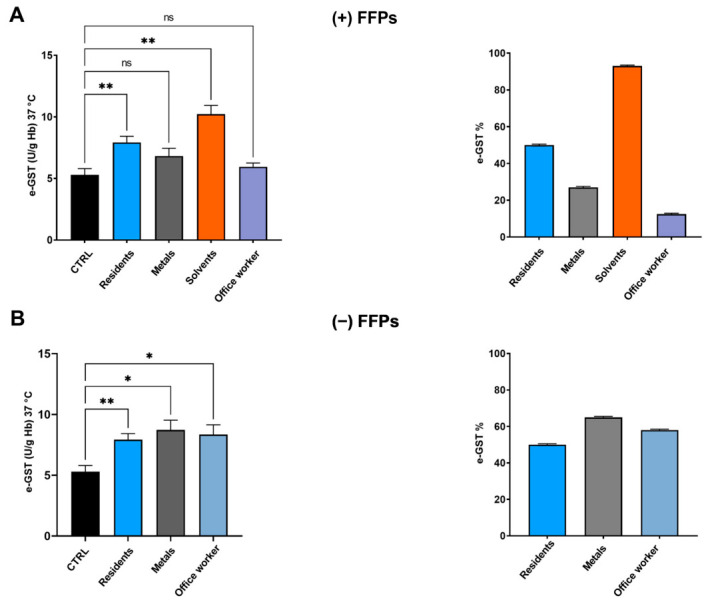
e-GST activity in male residents and foundry workers divided by job type and FFPs usage. (**A**) Left Panel: e-GST enzyme activity of the control group (black), residents (*n* = 13, light blue), workers using FFPs exposed to metals (*n* = 11, gray) or solvents (*n* = 5, orange), and office workers wearing FFPs (*n* = 6, light gray). Right Panel: the percentage increase of e-GST activity in residents (light blue), workers wearing FFPs exposed to metals/other materials (gray) or solvents (orange), and office workers wearing FFPs (light gray) compared to the control (5.3 U/g Hb). (**B**) Left Panel: e-GST enzyme activity of the control group (black), residents (*n* = 13, light blue), workers not using FFPs exposed to metals (*n* = 6, gray) and office workers (*n* = 6, light gray). Right Panel: the percentage increase in e-GST, in residents (light blue), workers not using FFPs exposed to metals, and office workers not wearing FFPs activity compared to the control (5.3 U/g Hb). Error bars represent SEM, ns (not significant), *p* ≤ 0.05 (*), *p* ≤ 0.01 (**).

**Figure 6:**
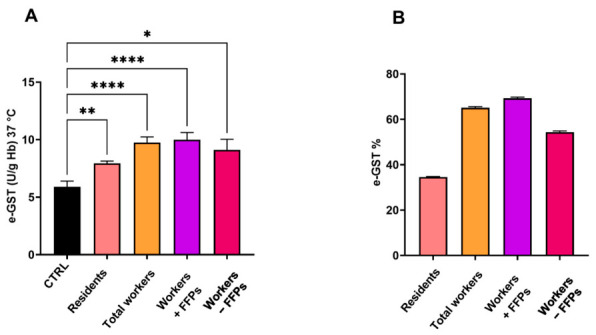
e-GST activity in female residents and foundry workers divided by job type and FFPs usage. (**A**) e-GST activity in the control group (black), residents (*n* = 15, pink), total female workers (*n* = 34, orange), workers using FFPs (*n* = 23, purple), and workers not using FFPs (*n* = 11, fuchsia). (**B**) Percentage increase of e-GST activity in residents (dark pink), total female workers (orange), workers using FFPs (*n* = 23, purple), and workers not using FFPs (*n* = 9, fuchsia) compared to the control (5.9 U/g Hb). Error bars represent SEM, *p* ≤ 0.05 (*), *p* ≤ 0.01 (**), *p* ≤ 0.0001 (****) compared to the control population.

However, the effectiveness of respiratory protection (FFP2/FFP3) became evident when data were stratified by job type. In particular, office workers and those exposed to metals showed an increase in e-GST activity, which was nearly half (+27%) of that observed in workers of the same category not using FFP masks (+65%). Conversely, this protective effect was notably absent in workers exposed to solvents, who showed the highest enzymatic increases (+90%) regardless of mask use (Figure 5 and Figure 6).

In summary, all these results indicate that solvent exposure represents a critical occupational factor, as standard FFPs offer limited protection against volatile chemical compounds (VOCs). Conversely, the protection of PPE is effective for workers exposed to metals, clay and sand, as well as for office workers. Indeed, this latter group exhibited an e-GST activity even lower than that of residents (Figure 5), suggesting that the use of PPE can offer superior protection against contaminants compared to the resident population, which does not use such devices.

### 3.4. e-GST Activity of Female Foundry Workers

Both female residents and workers showed significantly higher e-GST activities (7.9 ± 0.2 U/g Hb and 9.7 ± 0.5 U/g Hb, respectively) than the control group (5.9 ± 0.2 U/g Hb) (Figure 6) [17]. Therefore, similarly to what was observed for men, the e-GST activity of female workers and residents suggest elevated exposure to pollutants. However, contrary to what was found for men, the increase appears to be greater in the foundry environment (+65%) than in the residents’ area (+34%) (Figure 6).

**Figure 7:**
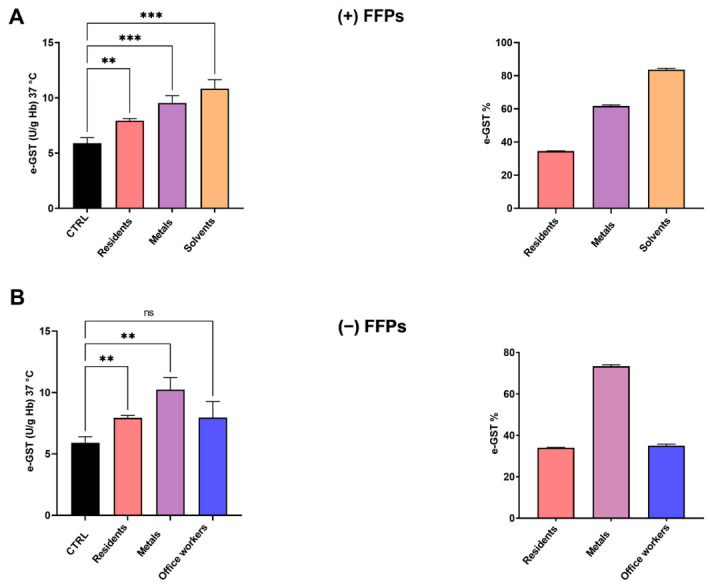
e-GST activity in female residents and foundry workers divided by job type and FFPs usage. (**A**) Left Panel: e-GST enzyme activity of the control group (black), residents (*n* = 15, dark pink), workers using FFPs exposed to metals (*n* = 15, violet) or solvents (*n* = 8, sand). Right Panel: percentage increase in e-GST activity among residents (dark pink), workers using FFPs exposed to metals (violet) or solvents (sand) compared to the control (5.9 U/g Hb). (**B**) Left Panel: e-GST enzyme activity of the control group (black), residents (*n* = 15, dark pink), workers not using FFPs exposed to metals (*n* = 8, violet) and office workers not using FFPs (*n* = 3, blue). Right Panel: percentage increase in e-GST activity among residents (dark pink), workers not using FFPs exposed to metals (violet), and office workers not using FFPs (blue) compared to the control (5.9 U/g Hb). Error bars represent SEM, ns (not significant), *p* ≤ 0.01 (**), *p* ≤ 0.001 (***), compared to the control population.

Unexpectedly, the total number of female workers using FFPs display a e-GST activity higher than that of workers without FFPs. An explanation for this paradoxical evidence becomes clear when observing the e-GST activities in the different job environments (Figure 7).

As highlighted in Figure 7, female workers exposed to metals are only slightly protected by using FFPs (from 73% of increase in the absence to 60% in the presence). Moreover, despite all female workers exposed to solvents using FFPs, this protection device is unable to prevent contamination for them as well as men, leading to a dramatic increase in e-GST of +82%. Interestingly, unlike their male counterparts, female office employees did not wear protective masks as they work in an area isolated from manufacturing zones. Their lack of exposure to hazardous materials is confirmed by e-GST activity, which is consistent with that of the resident population. In conclusion, these findings highlight a significant environmental risk in the manufacturing areas of women and confirm that the standard PPE fails to protect against solvent exposure.

### 3.5. e-GST Activity in Male and Female Ceramic Workers

**Figure 8:**
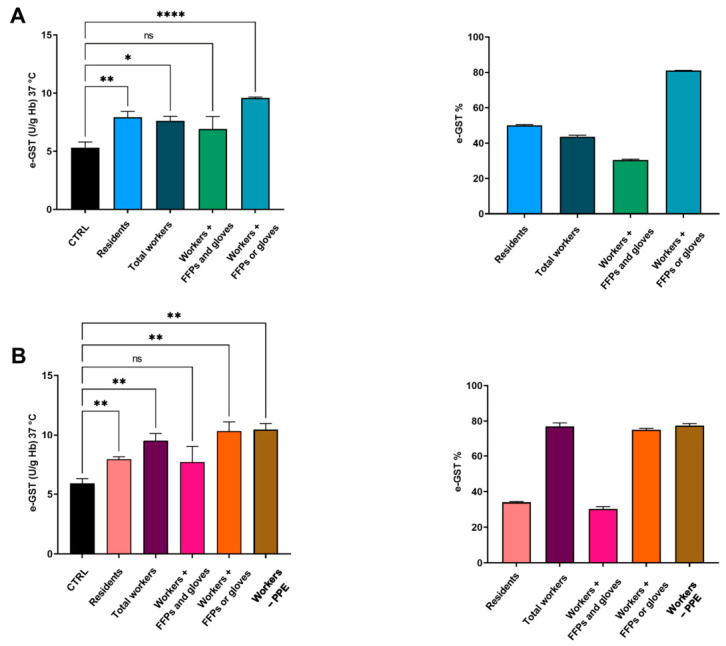
e-GST activity in male and female ceramists, divided by PPE type. (**A**) Left Panel: e-GST enzyme activity in the male control group (black), male residents (*n* = 13, light blue), male total workers (*n* = 7, dark blue), male workers using FFPs and gloves (*n* = 5, green), and male workers using FFPs or gloves (*n* = 2, cyan). Right Panel: the percentage increase in e-GST activity among male residents (light blue), male total workers (dark blue), male workers using FFPs and gloves (green), and male workers using FFPs or gloves (cyan) compared to the male control (5.3 U/g Hb). (**B**) Left Panel: e-GST enzyme activity in the female control group (black), female residents (*n* = 15, pink), female total workers (*n* = 12 violet), female workers using FFPs and gloves (*n* = 4, fuchsia), female workers using FFPs or gloves (*n* = 5, orange), and female workers not using PPE (*n* = 3, brown). Right Panel: percentage increase in e-GST activity among female residents (pink), female total workers (violet), female workers using FFPs and gloves (fuchsia), female workers using FFPs or gloves (orange), and female workers not using PPE (brown) compared to the female control (5.9 U/g Hb). Error bars represent SEM, ns (not significant), *p* ≤ 0.05 (*), *p* ≤ 0.01 (**), *p* ≤ 0.0001 (****) compared to the control population.

The mean e-GST activity for male ceramic workers enrolled in this study is similar to that of the resident population, showing an increase of +44% compared to the control. It should be noted that the type of exposure concerns only glaze, clay, and paints. FFPs and/or gloves are used by all workers (Figure 8A). Interestingly, the use of gloves or FFPs alone is not sufficient in guaranteeing a normal level of e-GST activity. In fact, ceramists who use only one PPE show an increase in e-GST of +80% (Figure 8A). In contrast, those using both gloves and FFPs showed a significantly lower increase of only +30%, a value even lower than that of the e-GST resident population.

Regarding the female workers, e-GST variation followed a similar but more pronounced pattern. Indeed, this group exhibited an increase in e-GST activity of about +60%, a value approximately double that of female residents (Figure 8B). This discrepancy does not lie in the type of working material, which is consistent with that handled by men ceramists, but rather in the level of PPE use. Notably, while all men wear at least one PPE, the 25% of women did not use any protective device, driving up the global mean of e-GST. However, as observed for male coworkers, the combined use of FFPs and gloves offers effective protection, reducing their e-GST activity to below that of residents. Conversely, the absence of one or both protective devices had a dramatic impact, leading to a substantial increase in e-GST of about +78% (Figure 8B).

Thus, these results underline the importance of additional PPE in specific occupational working areas, where contamination is not strictly linked to inhalation but also to the dermal absorption. Indeed, contact with paints is the main suspect for the increased e-GST activity observed.

### 3.6. Catholic Convent near the Foundry

**Figure 9:**
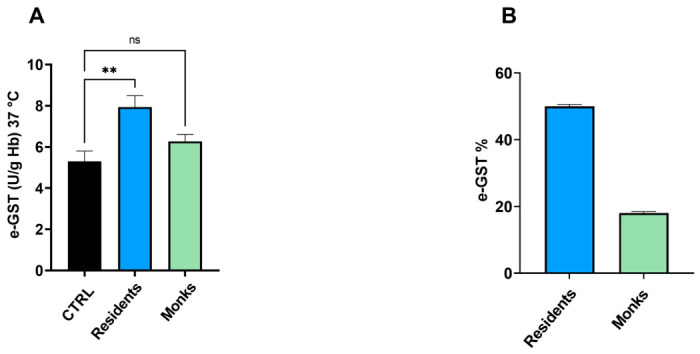
e-GST activity in male monks. (**A**) e-GST enzyme activity in the control group (black), residents (*n* = 15, light blue ), and monks (*n* = 14, light green). (**B**) Percentage increase in e-GST activity among residents (*n* = 15, light blue) and monks (*n* = 14, light green) compared to the control (5.3 U/g Hb). Error bars represent the SEM, ns (not significant), *p* ≤ 0.01 (**), compared to the control population.

The Catholic convent is located in close proximity to the foundry examined in the present study. Interestingly, all monks (*n* = 14) exhibited e-GST values significantly lower than those of resident population (+18% increase vs. +48% in residents) (Figure 9A,B). This notable divergence in e-GST activity cannot be easily explained, as no major dietary variations were reported between the two groups, and none of monks utilized protective devices. While these preliminary results are provocative, they must be interpreted with caution due to the small samples size (*n* = 14). Several confounding factors, which were not controlled for this study (i.e., different indoor ventilation patterns within the monastery, or distinct physical activity levels), might contribute to this observation. Rather than establishing a direct causal link between an ascetic lifestyle and reduced pollution impact, these data should be considered a preliminary observation that warrants further investigation into how micro-environments and lifestyle choices may modulate biomarker expression.

## 4. Discussion

A preliminary study enabled us to establish, for the first time, optimal blood storage conditions to preserve original e-GST activity. These findings are particularly relevant for future epidemiological studies utilizing e-GST as a biomarker. Specifically, whole blood maintains stability at 4 °C and 25 °C for up to three days, and at −80 °C for up to thirty days. Conversely, samples should never be stored at −20 °C, as an irreversible and progressive loss of affinity for GSH occurs within 24 h. Through the present study, all blood samples of foundry and ceramic workers, as well as of residents, were stored at +4 °C and analyzed for e-GST and HSAox within 24 h. The almost-normal HSAox levels in all workers of the foundry and ceramic compartments, as well as in the resident population, could suggest the absence of any oxidative stress. However, further studies investigating other biomarkers, such as lipid peroxidation (MDA) or DNA damage (8-OHdG), would be necessary to provide a comprehensive assessment of the environmental impact on the subjects’ redox balance. On the other hand, results concerning e-GST activity allow for some interesting observations. First, similar alteration of the activity of this enzyme in both the resident population and in ceramic /foundry workers could suggest common and similar pollutants. Looking at the different exposure levels of the workers, it is evident that solvent exposure in the foundry may be related to the highest increase in e-GST activity. Furthermore, FFP masks offered only negligible protection in certain areas, consistent with the known technical limitations of these devices in filtering VOCs [23]. Based on the observed e-GST variations across different sectors and the protective effects of FPP2/FPP3 masks, our data suggest that water and food are unlikely to be the primary source of contamination. For instance, the reduction in e-GST expression among masked office workers to levels below those of residents would be difficult to explain if the sources were dietary. It is plausible to hypothesize that contaminants are present both within and outside the foundry. Given their apparent filtration by FPP2/FPP3 masks, we speculate that these stressors may include airborne particles such as dust and/or metal fumes. However, as direct environmental or biological monitoring of these specific pollutants was not performed, these conclusions remain interpretative and require further analytical confirmation. An interesting observation about workers in the ceramic compartment is good protection from the combined use of gloves and FFPs but a relevant increase in e-GST in the absence of one of these protective devices. This points to the presence of unknown contaminants capable of being absorbed even by modest surfaces of epidermis. In this regard, paints would be strongly suspected. Indeed, both solvent-based coatings and water-based ones, which presents glycol ethers and additives poses, present inhalation risk. Furthermore, as noted by Mayer and coworkers regarding VOCs [24], the absence of even a single barrier, such as gloves, allows for rapid dermal absorption, which can explain the observed spike in e-GST activity.

Now, a brief discussion is necessary about the significance of an alteration of e-GST activity. As reported in many previous studies, an increase in this enzyme, which eliminates many toxic substances from the body, has been proposed as a warning sign of contamination both in humans and other mammalians like beef, sheep, and others [1,9]. We acknowledge that the present study does not provide direct quantification of environmental pollutants, their temporal fluctuations, or individual exposure doses. This lack of chemical–physical data represents a limitation in pinpointing the exact causative agents. However, the core objective of this research is to propose e-GST as a possible biological early-warning system. Unlike environmental sensors that measure a specific set of known chemicals, a functional biomarker, like e-GST, integrates the total biological impact of multiple, potentially unknown, stressors over time.

Although our findings clearly demonstrate a significant association between altered e-GST activity and populations working or living in areas characterized by high environmental pressure, some limitations must be acknowledged. This study does not provide direct quantitative measurements of specific environmental contaminants or dose–response relationships. Therefore, while e-GST represents a highly promising candidate biomarker of potential environmental exposure, further extensive validation studies integrating direct environmental monitoring are required to definitively establish its role as a robust indicator of pollution.

We again underline that e-GST could remember the behavior of white blood cells: their increased concentration in blood indicates a bacterial infection, but this signal does not identify the specific bacterium nor its dangerousness, both of which can be ascertained with subsequent investigations.

The e-GST analysis is intended to serve as a preliminary screening tool to prioritize environments for subsequent, more expensive, and time-consuming chemical tests, rather than replacing them. The observed enzymatic variations in our cohorts, even in the absence of detailed environmental mapping, suggest the potential sensitivity of this biomarker in reflecting biological stress under real-world exposure conditions. Similarly, the findings regarding the monastic community are particularly noteworthy. The fact that monks exhibit e-GST levels close to those of the control group, despite their proximity to the foundry, is presented here as a preliminary finding. These results could be a further indication of the interconnection between lifestyle and biological responses [25,26,27,28]. Although it is tempting to link these results to an ascetic lifestyle, the small sample size (*n* = 14) and potential confounding factors prevent any definitive causal conclusions at this stage. Future research incorporating direct environmental monitoring and larger cohorts will be essential to validate these preliminary observations and to better define the potential relationship between specific lifestyle factors and protection against environmental chemical stress.

## Figures and Tables

**Table 1:** Characteristics of the study population (Umbria Cohort).

Group	Total (N)	Male (*n*)	Male Mean Age (±SD)	Female (*n*)	Female Mean Age (±SD)
Control ^1^	400	260	43 ± 16	140	40 ± 17
Residents	28	13	64 ± 11	15	60 ± 9
Foundry Workers	66	34	49 ± 10	32	45 ± 7
Ceramists	19	10	44 ± 15	9	50 ± 8
Monks	14	14	45 ± 10	-	-

^1^ Control group referred to Ref. [13].

## Data Availability

The original contributions presented in this study are included in the article. Further inquiries can be directed to the corresponding author.

## Abbreviations

The following abbreviations are used in this manuscript:

e-GST	Erythrocyte glutathione transferase
SAox	Oxidized serum albumin
HSAox	Oxidized human serum albumin
HSAred	Reduced human serum albumin
GSH	Glutathione
CDNB	1-chloro-2,4-dinitrobenzene
EDTA	Ethylenediaminetetraacetic acid
DTNB	5,5′-dithiobis (2-nitrobenzoic acid)
PPE	Personal protective equipment
FFP	Filtering facepieces
FFP2	Filtering facepieces of class 2
FFP3	Filtering facepieces of class 3
VOCs	Volatile chemical compounds
